# Rat 50 kHz calls reflect graded tickling-induced positive emotion

**DOI:** 10.1016/j.cub.2020.08.038

**Published:** 2020-09-21

**Authors:** Justyna K. Hinchcliffe, Michael Mendl, Emma S.J. Robinson

**Affiliations:** 1 School of Physiology, Pharmacology and Neuroscience, University of Bristol, Biomedical Sciences Building, University Walk, Bristol BS8 1TD, UK; 2 Bristol Veterinary School, University of Bristol, Langford House, Langford BS40 5DU, UK

## Abstract

Positive animal emotion (affect) is a key component of good animal welfare [1] and plays an important role in stress-coping and resilience [2]. Methods for reliably inducing and measuring positive affect are critical, but both have been limited in availability. In rats, one promising way of inducing positive affective states is by human-simulated rough and tumble play or ‘tickling’ [3,4]. However, in humans tickling induces both pleasure and displeasure, and neither an established non-verbal indicator of positive affect, the Duchenne smile, nor laughter detects this variation [5,6]. Rats also show individual differences in response to tickling [7], and this variation needs to be readily quantified if we are to ensure that tickling is only implemented where it generates positive affect. Here, we use a validated and objective measure of affective valence, the affective bias test [8], to show that 50 kHz ultrasonic vocalizations provide a quantifiable and graded measure of positive affect that accurately reflects the positive state induced by this human–rat interaction.

## Teaser

Hinchcliffe and colleagues use the affective bias test to show that rats vary in the level of positive affect induced by simulated social play (‘tickling’), and that 50 kHz call rates provide a graded real-time measure of tickling-induced positive affect.

## Main Text

Reliable induction of positive affect in animals is critical if we are to successfully improve animal welfare [1] or generate model systems to investigate the putative benefits of such states [2]. In laboratory rats, ‘tickling’ is a widely advocated approach [3] (see: https://nc3rs.org.uk/news/tickling-rats-social-enrichment-improve-rodent-welfare). Tickling has beneficial effects but individuals vary in their response [7]. Blanket recommendations to implement tickling thus risk having unintended detrimental effects. This can be avoided if easy-to-use quantifiable and graded measures of positive affect allow accurate real-time monitoring of the response to tickling. Here, we ask whether, unlike Duchenne smiles and laughter in humans [5,6], 50 kHz rat ultrasonic vocalizations fulfil this role.

Answering this question requires a validated measure of affective valence that can quantify the strength of positive affect induced by tickling, thus providing a ground truth against which tickling-induced 50 kHz ultrasonic vocalizations can be gauged. Previous studies have been limited to measures of motivation such as approach to the tickler’s hand [4], or analysis of affective decision-making in rats who either do or don’t vocalize when tickled [9] but without investigating whether call rate reflects the strength of positive affect. Here we employ the affective bias test which has demonstrated the predicted affective valence for a wide range of pharmaceutical, hormonal, immune and environmental manipulations, yielding large effect sizes [8]. Moreover, dose-dependent bias data in drug studies indicate that it provides a highly sensitive, graded measure of the induced state [8].

Male Lister Hooded rats (n = 16)underwent an affective bias test, experiencing four independent training sessions (finding a food reward in a specific digging substrate), two after 30s tickling during which 50 kHz calls were measured (substrate A) and two under control conditions (substrate B), followed by a choice test (A vs B). A hand approach test [4] was carried out a week later (Supplemental Information).

**Figure 1:**
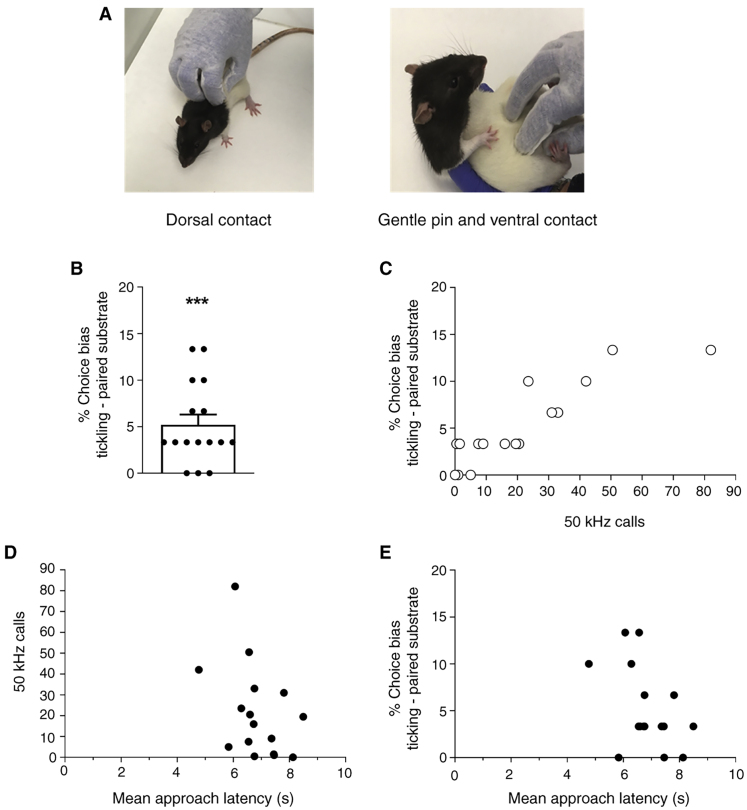
50 kHz calls emitted during tickling reflect positive affect as assessed by the ABT. (A) Example of the tickling stimulation method used as described by Panksepp and co-workers (Burgdorf *et al.*, 2008). Photographs by Justyna Hinchcliffe. (B) Rats show a positive bias towards the digging substrate experienced following tickling relative to that experienced following the control condition indicating induction of a relatively positive affective state by tickling at that time. Data shown as mean % choice bias ± SEM; one sample t-test against a null hypothesised mean of 0: t_15_ = 4.753, p = 0.0003 (^∗∗∗^on graph). (C) Scatter plot of relationship between % choice bias and the mean number of 50 kHz vocalisations emitted during tickling sessions prior to two substrate-reward training sessions; r = 0.8911, p < 0.0001. There was no correlation between mean approach latency to the experimenter’s hand and (D) the mean number of 50 kHz calls emitted during tickling sessions prior to two substrate-reward training sessions (r = -0.4157, p > 0.05) or (E) % choice bias (r = -0.4664, p > 0.05).

Rats showed a positive choice bias for substrate A (one sample t-test against null hypothesis of 0: t_15_ = 4.753, p = 0.0003, Figure 1B), indicating that tickling conferred greater reward-value to the digging experience and hence generated a more positive state than the control procedure. Moreover, there was a strong positive correlation between the choice bias score and the mean number of 50 kHz calls emitted during tickling sessions (Pearson’s correlation, r = 0.8911, p < 0.0001, Figure 1C; 50 kHz calls during individual tickling sessions also correlated strongly with choice bias, Supplemental Information), whilst no significant correlation was found between approach latency to the experimenter’s hand and either the number of 50 kHz calls emitted (r = -0.4157, p = 0.1093, Figure 1D) or the affective bias test choice bias score (r = -0.4664, p = 0.0686; Figure 1E).

Our findings show that, at a population level, tickling induced a more positive affective state. However, rats varied in how strongly they preferred the tickling-associated substrate (Figure 1B), just as drug dosage affected preference strength in previous affective bias test studies [8], implying that tickling induces stronger positive affect in some individuals than others. Therefore, not all rats like to be tickled [7] and when employing tickling to enhance affect and welfare, care should be taken to identify these animals. To this end, we show that 50 kHz ultrasonic vocalizations provide a real-time indicator of (tickling-induced) positive affect and, importantly, that the rate of calling reflects how positively valenced the state is (Figure 1C). 50 kHz ultrasonic vocalizations could thus be used to monitor the affective state induced by tickling. Latency to approach the experimenter’s hand did not reflect the strength of positive affect induced by tickling (Figures 1D,E; Supplemental Information), suggesting that interaction with the handler alone is not related to affective state and/or that hand approach is not a particularly sensitive measure of positive affect.

Overall, our findings demonstrate that 50 kHz USVs provide an easy-to-use, graded, and real-time measure of positive affect in response to a short-term event (tickling). Our results support the use of tickling to induce positive affect and welfare in rats, whilst confirming that rats, like humans, vary in how rewarding they find it. They also indicate that 50 kHz vocalisations may not be as closely related to tickling-induced human laughter as previously suggested. Unlike human smiles and laughter [5,6], 50 kHz vocalisations directly reflect the animal’s affective experience when being tickled. We suggest that future studies should use the affective bias test to investigate whether this generalises to other contexts in which 50 kHz vocalisations have been recorded *e.g*. juvenile play, mating, aggression [3]. Because the affective bias test is sensitive to both positive and negative affective states [8], hence addressing a long-standing challenge in studies of animal affect, and especially positive emotion, of establishing a ground truth state against which methods for inducing and measuring affect can be validated, it could also be used to investigate whether rat 22 kHz alarm calls provide a similarly graded measure of negative affective state. Furthermore, it could be implemented in other species to determine whether, as in rats, vocalisations can be veridical signals of affective state or, as in humans and possibly other great apes [10], they also reflect the influence of other social factors.

## Supplemental Information

Document S1. Experimental Procedures and One Figure

Data S1. Experimental counterbalancing, pellets consumed during the ABT test, and behaviour in the hand approach test (A) Counterbalanced allocation of rats to substrates and tickling / control treatments. (B) Pellets consumed from each substrate bowl during the ABT choice test. (C) Number of trials in hand approach test in which rats did or did not approach the hand within 10s, and mean approach latency either excluding non-approach data or including it coded as 10s.
