# Supplementary material for: Rat 50 kHz calls reflect graded tickling-induced positive emotion
Source: Curr Biol. 2020 Sep 21;30(18):R1034–5. doi: 10.1016/j.cub.2020.08.038 (PMC7533284; doi:10.1016/j.cub.2020.08.038)
Supplement: Document S1. Experimental Procedures and One Figure [file mmc1.pdf]

Supplemental information

Rat 50 kHz calls reflect graded tickling-induced positive emotion

Justyna K. Hinchcliffe, Michael Mendl and Emma S.J. Robinson

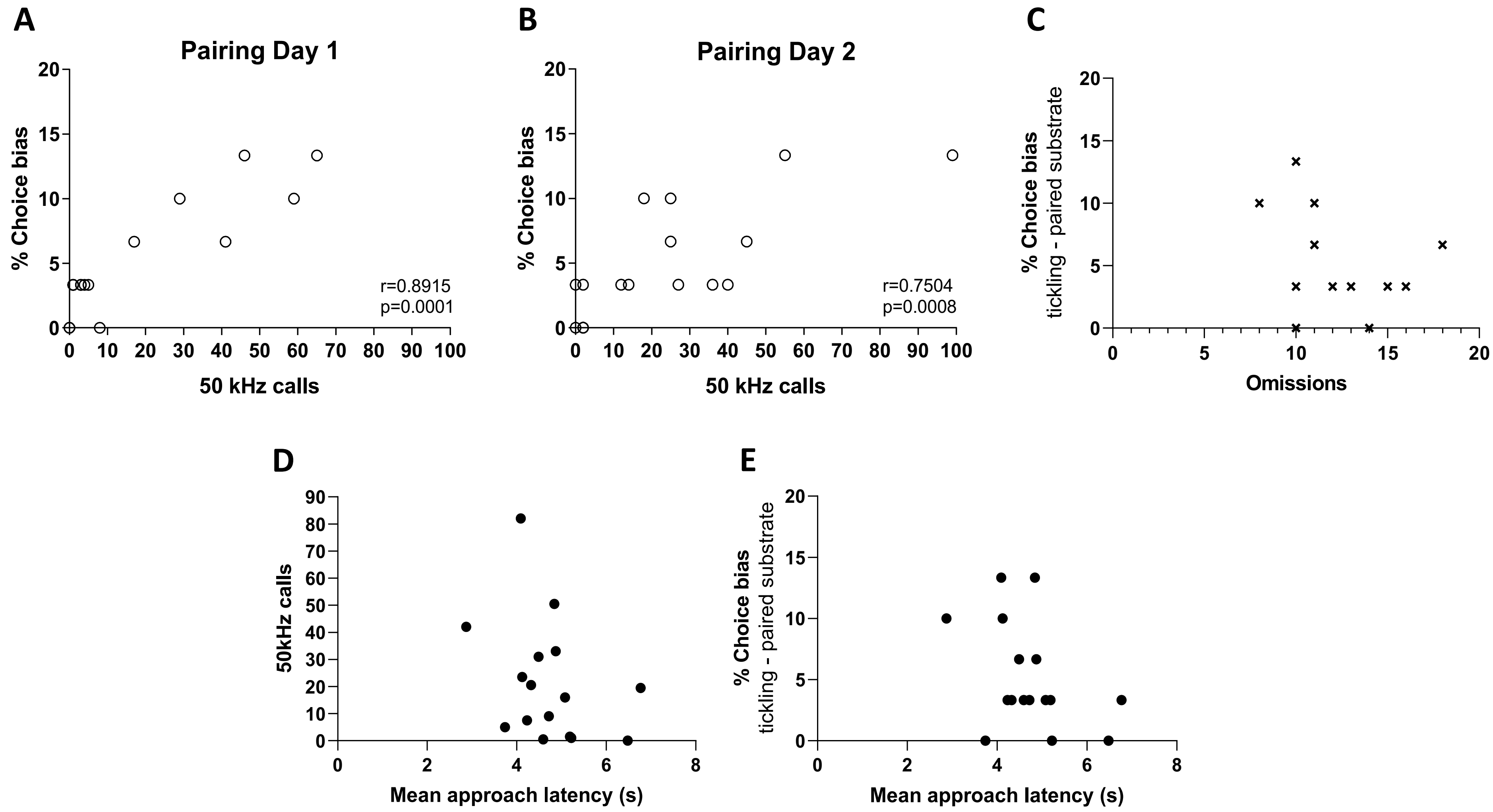

Figure S1. Relationships between ABT choice bias, USV call rate during tickling, and behaviour during the hand approach test

(A,B) Scatterplots of the relationship between % choice bias in the ABT and the number of 50kHz vocalisations emitted during the individual tickling sessions prior to two substrate-reward ABT pairing sessions (pairing day 1: r=0.8915, p=0.0001; pairing day 2: r=0.7504, p=0.0008). (C) Scatterplot of the relationship between % choice bias and the mean number of omissions during the hand approach test (r=-0.4111, p=0.1136). (D,E) Scatterplots of the relationship between mean approach latency to experimenter's hand (data exclude omission trials) and (D) the mean number of 50kHz calls emitted during tickling sessions prior to two substrate-reward training sessions (r=-0.3337, p=0.2065), or (E) choice bias score in the ABT (r=-0.4130, p=0.1119).

Supplemental Experimental Procedures

Animals and housing

The study used 16 male Lister Hooded rats (Harlan, UK) weighing around 300-350g at the start of training. Sample size was based on our previous Affective Bias Test (ABT) studies and meta-analysis which suggested a large effect size for the reward-induced positive bias in Lister Hooded rats [S1]. All animals were pair-housed in enriched laboratory cages (55x35x21cm) with sawdust, paper bedding, cotton rope, cardboard tubes and red Perspex houses (30x17x10cm), in temperature-controlled conditions (21±1°C) and under a 12:12h reverse light–dark cycle (lights off at 08:00h). Rats were food restricted to approximately 90% of their free feeding weights matched to the normal growth curve (~18 g of food per rat/day laboratory chow (Purina, UK)) and provided with *ad libitum* water. The behavioural procedures and testing were performed during the animals' active phase between 09:00h and 17:00h. All experimental procedures were conducted in accordance with the UK Animals (Scientific Procedures) Act 1986 and were approved by the University of Bristol Animal Welfare and Ethical Review Body and UK Home Office (PPL number P9B6A09A1).

Affective Bias Test (ABT)

General protocol

The ABT testing was carried out in a perspex arena (40x40cm) with two ceramic bowls (Ø 10cm) and a trio of digging substrates (reward-paired substrates - 'A' or 'B' versus unrewarded substrate - 'C', matched for digging effort and counter-balanced across subjects). The training and testing for the ABT were similar to those previously described [S1-S4]. Prior to ABT training animals underwent one habituation session to the ABT arena (without bowls, substrate or reward); rats were individually placed into the arena and allowed to explore for 10min. Further training consisted of three digging training sessions (20 trials per session) with a bowl filled with increasing amounts of digging substrate (sawdust) and a food reward (45mg purified rodent tablets, Test Diet, Sandown Scientific, UK). On the first day of digging training, each rat was placed in the arena and given 30s to approach and explore the empty bowl (without substrate) containing two pellets per trial. When the pellets were found and consumed, the trial was completed, and the rat was removed from the arena and the pellets were replenished in the bowl. During the next digging training session, each rat was given 30s to explore the bowl and start digging for a single pellet buried within 1 cm of sawdust. Following 20 trials in which the pellet was found and eaten, each rat was moved onto the final training session in which a single pellet was buried within 2 cm of sawdust. Once each animal was able to find a pellet within 30s on 10 consecutive trials (within a maximum 20 trials), the digging training was complete.

Animals then underwent a discrimination session allowing them to explore two bowls with two novel digging substrates (reward-paired substrate with single pellet versus unrewarded substrate). On

each trial, the animal was individually placed in front of the two bowls. Once the animal made a choice by starting to dig in one bowl, the other bowl was removed by the experimenter. Choice of the reward-paired substrate was marked as a 'correct' trial, digging in the 'blank' substrate was classified as an 'incorrect' trial and if an animal failed to approach and explore the bowls within 30s, the trial was recorded as an 'omission'. Trials were continued until the rat attained six consecutive correct choices for the reward-paired substrate. The discrimination session allowed us to confirm that the animals could achieve our learning criterion of six consecutive correct trials in less than 20 trials.

Tickling study

The tickling experiment was composed of four *pairing sessions* (one per day) followed by a *choice test* on the fifth day of the same week. During pairing sessions, each trial involved presenting the rat with a choice between two bowls containing two different digging substrates (e.g. pink fur fabric, purple polyester fabric, pompoms), one of which was reward-paired (substrate A or B, counter-balanced across subjects and manipulation, Data S1A) and contained a single 45mg food pellet, and the other of which was unrewarded (substrate C). Substrate C was kept the same for all four pairing sessions and a 45mg pellet was crushed into the bowl and mixed within the substrate, to prevent choices based on odour. One of substrates A or B was presented during pairing sessions on days 1 and 3, and the other was presented on days 2 and 4, with order counterbalanced across subjects (see Data S1A). Prior to substrate A (or B) versus C sessions, rats were exposed to 30s of tickling and the mean number of 50kHz USVs emitted was recorded. The control condition prior to B (or A) versus C sessions was to remain in the home cage. The aim was to train an association between the tickling-induced state and digging in substrate A (or B) for a rewarding food pellet, and a control state and digging in substrate B (or A) for the same food reward. A pairing session was completed when the rat chose the rewarded substrate on six consecutive trials. All factors (i.e. bowl location, substrates paired with tickling manipulation, pairing sessions) were fully counterbalanced. The number of trials to reach criterion (six consecutive correct choices for the reward-paired substrate), and latency to dig were recorded for each animal.

The effect of tickling was then quantified during the choice test on day 5 when the two previously rewarded substrates (A and B) were presented at the same time for 30 trials. Preference for the tickling-paired substrate indicated that the tickling-induced state conferred greater reward-value to the digging experience and hence was more positive than the control state, whilst preference for the control-paired substrate indicated the opposite. A choice bias score was calculated as detailed in the 'Data analysis' section below. As in previous ABT studies, in order to keep rats motivated to continue choosing without providing new associative information, a single 45mg food pellet was placed in either bowl using a random schedule with a probability of one in three so that rats randomly received a reward (i.e. substrate A contained a pellet on 10 of the 30 trials, and likewise for substrate

B; on no trials were both bowls baited). Both bowls also had a pellet crushed and placed in the substrate to reduce the likelihood of the animal using odour to find the reward. The animals' choices and latency to dig were recorded.

Tickling procedure

A modified version of the Panksepp tickling stimulation procedure was used [S5, S6]. Prior to the experiment animals underwent three tickling habituation sessions. In all sessions, rats were individually transferred to the experimental room and placed into a test box (34x22x26.5cm white rectangular box) where tickling stimulation was administered for 30s by the experimenter (JKH) wearing cotton gloves (see Figure 1A in main article). The procedure mimics the rough-and-tumble play of rats, consisting of repeated vigorous finger movement across the dorsal body surface of the animal followed by rapid flipping of the rat on to its back and tickling the abdomen as it is gently pushed against the floor of test box, followed by release [S5, S6]. 30s tickling sessions were subsequently carried out in the test box prior to ABT pairing sessions involving the tickling-associated substrate as described above.

Recording and analysis of ultrasonic vocalisations (USVs)

USV calls were recorded during the two pairing days by using a high frequency microphone (2- to 200- kHz range, CM16/CMPA UltraSoundGate Condenser Microphone, Avisoft Bioacoustics, Germany) suspended 15 cm above the floor of the test box and connected to a computer via an ultrasound recording interface (116H UltraSoundGate, Avisoft Bioacoustics, Germany), and acoustic data were displayed in real time by Avisoft RECORDER USGH (version 4.2., Avisoft Bioacoustics, Germany). To evaluate ultrasonic vocalization data spectrograms were generated by SASLab Pro (version 5.2., Avisoft Bioacoustics, Germany) and criteria for call identification was set at frequencies between 35-80 kHz and durations of 30-50ms for frequency-modulated 50kHz calls, and frequencies between 18-32 kHz and durations of 300-4000ms for 22 kHz calls [S7]. Low- (below 18 kHz) and high-pass (above 80kHz) filters were used to reduce background noise outside the relevant frequency band. The numbers of 50kHz calls and 22kHz calls were scored by an experienced observer (JKH) without prior knowledge of the ABT results. All USVs recorded were found to be in the 50kHz range with no 22kHz calls detected. USV data collected during the tickling manipulations were not analysed until after the ABT was completed, and with the researcher blinded to the results of the ABT choice test.

Hand approach test

The ABT involves extensive human contact due to handling of the animals during each trial. Furthermore, during ABT training days when tickling is implemented, human-rat interaction in general (e.g. including handling and transfer to the tickling test box) is greater than on control days.

Therefore to check whether any observed affective bias was related to individual differences in rat responses to human contact per se, as well as to any specific effects of tickling, we carried out a modified hand approach test in the week following the ABT experiment [S8-S10]. Testing was conducted in the ABT arena. On each trial, each rat was individually placed in one of four corners of the arena and the experimenter then placed one gloved hand in one of the remaining corners. All animals underwent 30 trials with 5s intervals between them (a randomised sequence for each rat of 12 trials with the hand placed in a corner located diagonally from the rat's start position, and 18 trials with the hand placed in a non-diagonally located corner). The latency for the rat to approach from a distance of 40-50cm to touch the experimenter's hand was measured using a stopwatch. If a rat failed to approach the hand within 10s, the trial was scored as an 'omission' and the animal was held for 5s before starting the next trial. It is worth noting that unlike in the previous study by Burgdorf and Panksepp [S8], the animals used for the ABT had undergone extensive handling and habituation as well as ABT training.

Data analysis

Data were analysed using GraphPad Prism 8.4 (GraphPad Software, USA). Choice bias score was calculated as the number of choices made for the tickling-paired substrate divided by the total number of trials multiplied by 100 to give a percentage value. A value of 50 was then subtracted to give a score where a choice bias towards the tickling-paired substrate gave a positive value and a bias towards the control-paired substrate gave a negative value. Choice bias scores were analysed utilising a one-sample t-test against a null hypothesised mean of 0% choice bias. For each animal, mean latency to dig and trials to criterion during ABT pairing sessions were analysed using a paired t-test comparing control versus tickling-manipulation sessions, in order to check for any non-specific effects of treatment. Pearson's correlations were performed to investigate relationships between choice bias in the ABT test, approach latencies in the hand approach test, and 50kHz USVS during the tickling sessions.

Supplemental Results

Relationship between calls emitted during each tickling session and choice bias in the ABT

In addition to the mean data for 50kHz calls we also compared data from the choice test with USV from each of the pairing sessions independently. There was a strong positive correlation between each rat's choice bias score in the ABT and the number of 50kHz calls that it made during each of the two tickling sessions (session 1: $r=0.8915$, $p=0.0001$; session 2: $r=0.7504$, $p=0.0008$; Figures S1A,B).

Pellet consumption data in the ABT choice test

Data S1B shows the number of pellets consumed from each substrate bowl during the 30 trials of the ABT choice test. This is used to check whether adding a single pellet to each substrate bowl with a probability of 1 in 3 (designed to keep rats motivated to continue choosing without providing new associative information) inadvertently results in them making choices based on pellet location. If so, we would expect them to earn well over 10 pellets per session (chance given that on no trials are both bowls baited). However, the data show that the mean number of pellets earned is 10.81 indicating that this is not the case, likely because the addition of crushed pellets to each bowl minimises the possibility of rats using odour cues to guide their choices.

Hand approach test

Data S1C shows the number of trials on which an approach to the hand was made within 10s, the number of trials on which an approach was not made (omissions), and the mean approach latency (both excluding omissions and including them coded as 10s). There was no relationship between the number of omissions that a rat made during the hand approach test and its choice bias score in the ABT (r=-0.4111, p=0.1136; Figure S1C). When omissions were excluded from data analysis, there was no significant correlation between each rat's mean hand approach latency and either its mean 50kHz vocalizations during the tickling sessions (r=-0.3337, p=0.206; Figure S1D) or its choice bias score in the ABT (r=-0.4130, p=0.112; Figure S1E). The same was found when omissions were included in the analysis and assigned a 10s value (see main paper, Figures 1D,E).

Choice latency and trials to criterion

No differences were observed for latency or trials to criterion during the different types of pairing sessions (response latency (s): control (3.4±0.2) vs tickling (3.2±0.2), t_{15}=1.039, p=0.315; trials to criterion: control (7.0±0.2) vs tickling (6.6±0.1), t_{15}=1.321, p=0.206).

Supplemental References

S1. Hinchcliffe, J.K., Stuart, S.A., Mendl, M. and Robinson, E.S.J. (2017). Further validation of the affective bias test for predicting antidepressant and pro-depressant risk: effects of pharmacological and social manipulations in male and female rats. Psychopharmacol. *234*, 3105-3116.

S2. Stuart, S.A., Butler, P., Munafo, M.R., Nutt, D.J. and Robinson, E.S.J. (2013). A translational rodent assay of affective biases in depression and antidepressant therapy. Neuropsychopharm. *38*, 1625-1635.

S3. Stuart, S.A., Butler, P., Munafo, M.R., Nutt, D.J. and Robinson, E.S.J. (2015). Distinct neuropsychological mechanisms may explain delayed- versus rapid-onset antidepressant efficacy. Neuropsychopharm. *40*, 2165-2174.

S4. Stuart, S.A., Wood, C.M. and Robinson, E.S.J. (2017). Using the affective bias test to predict drug-induced negative affect: implications for drug safety. Br. J. Pharmacol. *174*, 3200-3210.

S5. Panksepp, J. and Burgdorf, J. (2000) 50-kHz chirping (laughter?) in response to conditioned and unconditioned tickle-induced reward in rats: effects of social housing and genetic variables. Behav. Brain Res. *115*, 25-38.

S6. Knutson, B., Burgdorf, J. and Panksepp, J. (2002). Ultrasonic vocalizations as indices of affective states in rats. Psych. Bull. *128*, 961–977.

S7. Burgdorf, J., Panksepp, J. and Moskal, J.R. (2011). Frequency-modulated 50 kHz ultrasonic vocalizations: a tool for uncovering the molecular substrates of positive affect. Neurosci. Biobehav. Rev. *35*, 1831-1836.

S8. Burgdorf, J. and Panksepp, J. (2001) Tickling induces reward in adolescent rats. Physiol. Behav. *72*, 167-173.

S9. Panksepp, J. and Burgdorf, J. (2003) "Laughing" rats and the evolutionary antecedents of human joy? Physiol. Behav. *79*, 533-47.

S10. Hurst, J.L. and West, R.S. (2010). Taming anxiety in laboratory mice. Nat. Methods. *7*, 825-826.
